# Contribution of Visible Surface Mold to Airborne Fungal Concentration as Assessed by Digital Image Quantification

**DOI:** 10.3390/pathogens10081032

**Published:** 2021-08-15

**Authors:** Chun-Chieh Tseng, Ning Huang, Chia-Jung Hsieh, Chien-Che Hung, Yue-Liang Leon Guo

**Affiliations:** 1 Department and Graduate Institute of Public Health, Tzu Chi University, Hualien 97004, Taiwan; tsengcc@mail.tcu.edu.tw (C.-C.T.); cjhsieh@mail.tcu.edu.tw (C.-J.H.); gavink23@gmail.com (C.-C.H.); 2 Institute of Environmental and Occupational Health Sciences, College of Public Health, National Taiwan University, Taipei 10617, Taiwan; r95841018@ntu.edu.tw; 3 Environmental and Occupational Medicine, National Taiwan University College of Medicine and NTU Hospital, Taipei 10617, Taiwan

**Keywords:** bioaerosol, fungi, surface, indoor air

## Abstract

The rapid monitoring of total fungi, including air and surface fungal profiling, is an important issue. Here, we applied air and surface sampling, combined with digital image quantification of surface mold spots, to evaluate the contribution of surface fungi to airborne fungal concentrations. *Cladosporium*, *Penicillium*, *Aspergillus*, and yeast often appeared in the air or on wall surfaces during sampling. The indoor/outdoor concentration ratios (I/O ratios) demonstrated that the airborne concentrations of commonly found fungal genera outdoors were higher than those indoors (median I/O ratio = 0.65–0.91), excluding those of *Penicillium* and yeast. Additionally, the surface density (fungal concentration/area) of individual fungi showed no significant correlation with the airborne concentration, excluding that of *Geotrichum*. However, if a higher surface ratio (>0.00031) of mold spots appeared in the total area of an indoor environment, then the concentrations of *Aspergillus* and *Geotrichum* in the air increased significantly. Our results demonstrated that the airborne concentration of indoor fungi is significantly correlated with the outdoor concentration. A higher density of surface fungi does not necessarily contribute to a high fungal concentration in the air. In contrast to fungal density, quantification of the surface fungal area is recommended to assess the risk of surface fungi propelling into the air.

## 1. Introduction

Fungal spores are ubiquitous in indoor environments and often present as mixtures of fungal genera. Although fungal types and concentrations in nonproblematic indoor environments are considered to originate primarily from outdoor air [1,2,3], mold contamination on wall surfaces may also affect fungal concentrations in indoor air [4]. Especially in cases when water damage or moisture accumulation appears on walls, molds that rapidly colonize surfaces may propel into the indoor air with airflow or external force and further affect the air quality [4]. To date, several studies have demonstrated the relationship between fungal exposure and allergic symptoms, including night cough, wheezing, dyspnea, and allergic rhinitis [5,6]. Therefore, an efficient fungus monitoring method is needed to explore the total amount of human exposure to fungi; such a method should consider the fungal profile both in the air and on the surfaces of indoor environments.

To identify the potential fungal reservoir, comparisons between indoor/outdoor fungal concentrations in the air have been used to document the presence of fungal contamination. In Taiwan, 2.9 to 31.4% of residential environments have indoor/outdoor concentration ratios (I/O ratios) higher than one for specific fungal species, including *Aspergillus* spp., *Penicillium* spp., *Cladosporium* spp., *Alternaria* spp., and yeast [7]. Thus, the concentration of fungi in indoor air may not originate solely from outdoor air. The potential fungal reservoir may be derived from wall surfaces or house dust [8,9].

At present, although different sampling and analysis methods are available, there are still no widely acceptable standard protocols for measuring fungi in indoor environments. Sampling methods can be divided into air and surface sampling. Air sampling is commonly used to determine the fungal concentration and composition in the air by an active/passive sampling apparatus [10]. Surface sampling has been applied to identify localized surface mold contamination problems with cotton swabs, direct sampling with tapes, or Rodac (Replicating Organism Direct Agar Contact) plates [4,10]. Although a previous study suggested that air and surface sampling should be combined to obtain a robust result, it was also indicated that there was no strong correlation between the results obtained from air and surface testing [10]. While these results may be related to the different sampling methods used, they may also imply that the correlation between air and surface fungi is species dependent, since no species identification was conducted in this study [10].

In practice, almost all surface sampling addresses only the mold density on the surface per area (e.g., CFU/cm^2^) [11], and very limited studies have quantified the area of mold growth on surfaces. The discovery of visible mold growth on a surface warrants a recommendation for cleanup and investigation of the possible reason for the growth [1]. However, some difficulties in correctly determining the mold surface area have to be overcome, especially when the surfaces are small or have a nonuniform shape. Conversely, mold spores can be divided into wet spores and dry spores. A previous study indicated that dry spores, including those of *Penicillium*, *Aspergillus*, and *Cladosporium*, can be easily propelled into the air, whereas wet spores, such as those of *Stachybotrys*, *Acremonium*, and *Trichoderma*, are considered to produce mucilaginous masses that stick firmly to surfaces [4]. Consequently, the ability of fungi to float from the surface into the air could be related to the hydrophilic or hydrophobic properties of spores. In addition, relative humidity has also been found to directly influence the release of conidia from conidiophores [2].

To understand the contribution of wall fungi to the concentration of airborne fungi in residential environments, the aims of this investigation were (1) to evaluate the correlation between the environmental factors and airborne/surface fungi in the residential environment; (2) to determine the correlation among the fungal concentrations recovered from outdoor, indoor, and surface sampling; (3) to apply a simple digital image quantification method for calculating the total mold surface area and then use this indicator to evaluate the surface mold ratio, with a greater emphasis on airborne fungal concentrations.

## 2. Results

### 2.1. Temperature, Relative Humidity, and Wind Speed in Indoor Environments

The average atmospheric temperature and relative humidity (RH) were 30.0 °C (from 25.1 to 36.2 °C) and 67.6% (from 45.3 to 88.8%) throughout the sampling periods, respectively. In addition, the average surface temperature was 31.4 °C (from 27.82 to 36.8 °C), and the mean surface RH was 68.2% (from 37.9% to 89.93%). Two other environmental parameters, the CO_2_ concentrations (average = 632 ppm; from 400 to 1200 ppm) and wind speed (average = 0.2 m/s; from 0 to 1.45 m/s), were also measured.

### 2.2. Concentrations of Airborne Fungi in Indoor and Outdoor Air

**Figure 1:**
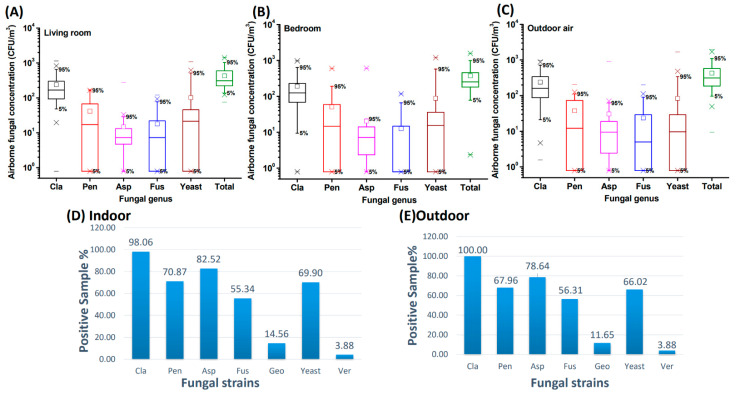
The concentration (CFU/m^3^) distribution of different fungal genera in the indoor air of living rooms (**A**) and bedrooms (**B**) and in outdoor air (**C**). Figures (**D**,**E**) show the % positive samples having the respective fungal genera in the indoor air (**D**) and outdoor air (**E**) of 103 household samples. Cla = *Cladosporium*; Pen = *Penicillium*; Asp = *Aspergillus*; Fus = *Fusarium*; Geo = *Geotrichum*; Ver = *Verticillium*. The square □ is the average value of the individual fungal genus.

Figure 1 shows the airborne fungal concentration in the indoor air (including the living room and bedroom air; Figure 1A,B) and outdoor air (Figure 1C). There was no significant difference in the total concentration of airborne fungi between indoor (median = 278.0 CFU/m^3^) and outdoor air (median = 313.3 CFU/m^3^; *p* = 0.309). However, the fungal concentrations in the indoor air were significantly lower than those in the outdoor air for *Cladosporium* (*p* = 0.038), *Aspergillus* (*p* = 0.003), and *Fusarium* (*p* = 0.024). Only the concentration of yeast was found to be significantly higher in indoor air than in outdoor air (*p* = 0.012). Due to the low detection rates of *Geotrichum* (12–15%) and *Verticillium* (3.88%), the concentrations of these two fungi are not presented in Figure 1. Based on the distribution of fungal concentrations, the most predominant fungal genera, with higher concentrations, were *Cladosporium*, yeast, *Penicillium*, *Aspergillus,* and *Fusarium*, regardless of indoor or outdoor air. *Cladosporium* ranked first, with more than 98% of house samples containing *Cladosporium* (Figure 1D,E). In addition to *Cladosporium*, more than 65% of household air samples contained *Aspergillus*, *Penicillium*, and yeast.

**Figure 2:**
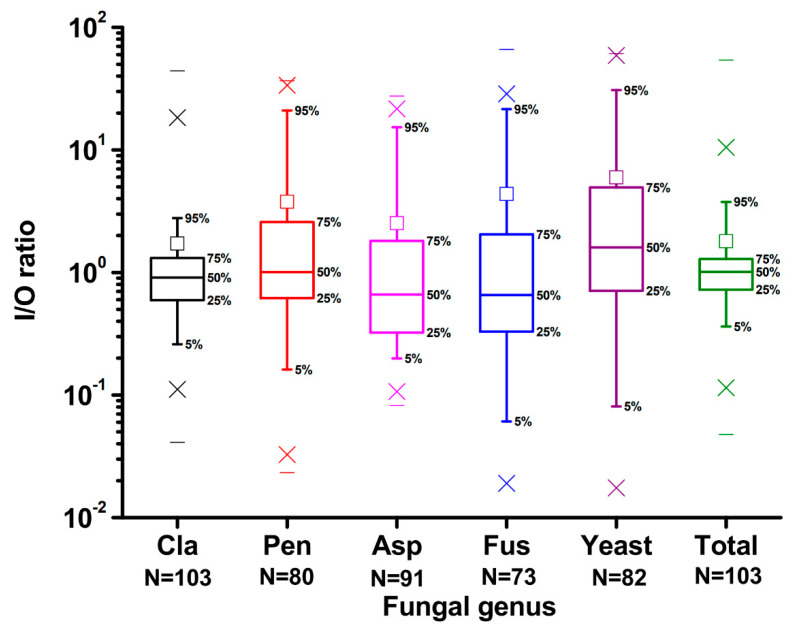
The indoor/outdoor concentration ratios (I/O ratios) for different fungal genera recovered from the air. The square □ above the box is the average value of the individual fungal genus. Cla = *Cladosporium*; Pen = *Penicillium*; Asp = *Aspergillus*; Fus = *Fusarium*; Total = total fungi.

To identify potential sources of biological contamination, the specific concentrations of indoor and outdoor airborne fungi have often been compared [7,12]. Consequently, we calculated the I/O ratio for almost all fungal genera recovered from the air (except for *Geotrichum* and *Verticillium*) (Figure 2). In addition, due to the low detection rate of *Geotrichum* and *Verticillium*, the I/O ratios of these two fungi are not presented in Figure 2. Furthermore, if the corresponding fungus was not detected in the airborne sample, then this sample was excluded from the I/O ratio calculation. Since the concentration of fungi in household air samples varied greatly, we used the median to represent the I/O ratio. Except for *Penicillium* (I/O = 1.002), yeast (I/O = 1.56), and total fungi (I/O = 1.01), the I/O ratios of the fungal genera recovered from the air were less than 1 (Figure 2).

### 2.3. Concentrations of Surface Fungi in Indoor Environments

**Figure 3:**
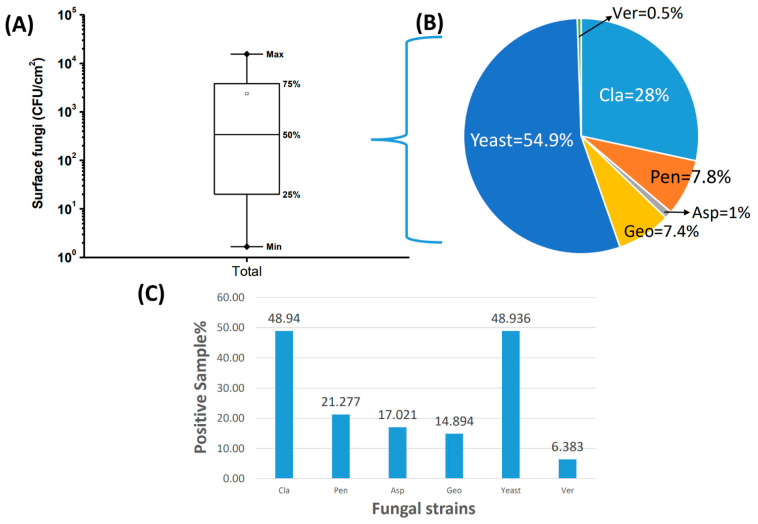
The distribution of the total fungal concentration (CFU/cm^2^) on the wall surface of household samples (**A**). The percentage shown in the pie chart (**B**) is the concentration distribution of individual fungal genera among the total surface fungi. The bar chart (**C**) shows the % positive samples having the respective fungal genera on household wall surfaces where visible mold spots were observed (*n* = 47). Cla = *Cladosporium*; Pen = *Penicillium*; Asp = *Aspergillus*; Geo = *Geotrichum*; Ver = *Verticillium*; Total = total fungi.

There were no visible mold spots on the surfaces of some resident environments (*n* = 56). Figure 3 shows the distribution of the surface fungal concentration in environments where visible mold spots were present (*n* = 47). Figure 3A shows that the median total fungal concentration on household wall surfaces was 340.0 CFU/cm^2^. Except for *Fusarium*, fungal genera that appeared on wall surfaces also appeared in the air. Because the detection rate of certain fungi was low (Figure 3C), Figure 3B does not use the arithmetic mean or median to represent the concentration of individual surface fungi; it is only presented in terms of the proportion of the respective fungal genus to the total fungal concentration. The most predominant fungal genera on the wall surface were *Cladosporium* and yeast. These two major fungal genera accounted for more than 80% of the total surface fungi, and their concentrations varied widely from N.D. (not detected) to 10,253.3 CFU/cm^2^. In addition, approximately 49% of the household samples contained *Cladosporium* and yeast (Figure 3C). Among household samples where mold spots appeared on the surface, 68% of the samples had only one fungal genus, 17% of the samples contained two genera of fungi, and only 14% of the identified samples contained more than three fungal genera. The diversity of fungal genera contained in the mold spots was lower than that in air samples.

### 2.4. Correlation among Environmental Parameters and Airborne Fungal Concentrations

As shown in Table 1, the correlation among the environmental parameters (temperature, atmospheric RH, wind speed, and CO_2_) and airborne fungal concentrations in the indoor environment was determined by Spearman’s rank correlation. *Cladosporium*, *Fusarium*, and *Verticillium* in indoor air were significantly correlated with temperature. In addition, the airborne concentrations of *Penicillium* were significantly related to high RH. However, *Fusarium* and *Verticillium* were negatively correlated with RH. Only *Verticillium* was correlated with wind speed. For CO_2_, all statistically significant correlations were negative correlations for the concentrations of airborne *Cladosporium*, *Aspergillus*, *Fusarium*, *Verticillium*, and total fungi.

### 2.5. Correlation among Environmental Parameters and Surface Fungal Density

The correlations among the environmental parameters (surface temperature, surface RH, wind speed, and CO_2_) and surface fungal concentrations in the indoor environment are shown in Table 2. Because *Fusarium* was not observed on the surfaces of indoor environments, no correlation result is shown for *Fusarium* in Table 2. On the environmental surfaces, no correlations were observed between fungi and temperature. Only *Geotrichum* and *Verticillium* were correlated with surface RH, and the total fungi were negatively correlated with wind speed. In addition, the surface densities of yeast and total fungi were significantly correlated with CO_2_ concentrations.

### 2.6. Correlation between the Concentration of Indoor and Outdoor Fungal Aerosols

The correlation between the concentration of indoor and outdoor fungal aerosols is shown in Table 3. From the perspective of total fungi, indoor and outdoor fungal aerosol concentrations showed a significant correlation. All known indoor concentrations of individual fungi were also significantly correlated (Spearman’s rank correlation coefficients ranged from 0.48 to 1; all *p* values < 0.01) with their outdoor concentrations. In addition, the indoor concentrations of *Cladosporium* were correlated with almost all the fungi (except for yeast) detected in indoor air (*p* values ranged from <0.01 to 0.019). Furthermore, the indoor yeast concentration was negatively correlated with almost all other indoor fungal concentrations (except for *Cladosporium* and *Fusarium*), although some correlations were not significant (*p* values ranged from <0.01 to 0.391).

### 2.7. Correlation between the Concentrations of Airborne and Surface Fungi in Indoor Environments

Table 4 shows the correlations between the concentrations of airborne and surface fungi in the indoor environments. Among all the surface fungi found, only *Geotrichum* exhibited airborne concentrations that were significantly correlated with its surface density (Spearman’s rank correlation coefficient = 0.420; *p* < 0.01). However, when only surface fungi were compared, the densities of almost all fungal genera were significantly correlated with each other (except for yeast), with Spearman’s rank correlation coefficients ranging from 0.30 to 0.70 (all *p* values < 0.01). For the surface yeast, the concentration was consistently non-significantly correlated with that of other fungal strains on the surface (*p* values ranged from 0.076 to 0.596).

### 2.8. Association among the Airborne Fungal Concentration, Fungal Surface Area Ratio, and Environmental Parameters

In Table 5, we divided the fungal surface ratio into three levels (0, 0–0.00031, and 0.00031–1), based on the median of our data (Supplementary Material). Next, we included three environmental factors that may affect the concentration of airborne fungi in the multiple linear regression model for analysis adjustment. Using the model, we found that if a higher surface area ratio (0.00031–1) appeared in the environment, the indoor air would present a significantly higher concentration of *Aspergillus* and *Geotrichum* aerosols than the reference group (surface ratio = 0). In addition, a dose-dependent response was also observed for *Aspergillus* and *Geotrichum*, and only the highest level 3 surface area ratio contributed to a significantly higher indoor air concentration (139% for *Aspergillus*, *p* = 0.005, and 85% for *Geotrichum*, *p* = 0.014) than the reference group. The level 2 surface ratio increased airborne *Verticillium* concentrations by 39.8% compared to the reference group. However, this correlation was not significant for *Verticillium* (*p* = 0.108).

## 3. Discussion

Fungal sampling in this study was conducted in summer. A number of studies have shown that the concentration of airborne fungi in summer is higher than that in winter [13,14,15]. Although the concentration of fungi found in southern Taiwan in winter was higher than that in summer [16], the sampling sites in this study were evenly distributed throughout Taiwan, and seasonal changes in airborne fungal concentrations were not the major focus of this study. In general, the temperature and humidity measured in previous similar fungal studies were within the measured range of our results [7,13,16]. In addition, only nine airborne samples exceeded 1000 ppm of CO_2_ concentration in the indoor air, which is the stipulated standard of Taiwan’s indoor air quality management act.

The most predominant airborne fungal genera in our study agreed with those in previous studies conducted in Taiwan [7,13], except for the difference in the predominant ranking of fungi. However, *Cladosporium*, *Penicillium*, and *Aspergillus* always appeared to be predominant in air sampling [17]. In addition, the airborne concentrations of these predominant fungal genera, including *Cladosporium*, yeast, *Penicillium*, *Fusarium*, and *Aspergillus*, were also similar to a previous study’s findings of 10^0^ to 10^2^ CFU/m^3^ [14]. *Cladosporium* usually dominated among all airborne fungi in indoor and outdoor air. Since *Cladosporium* is a well-known genus from the outdoor environment, a previous study demonstrated that the I/O ratio of *Cladosporium* might be less than 1 [18] (median = 0.91 in our study). Another fungal genus that may arise outdoors is *Penicillium*, which is associated with soil and plants. However, the I/O ratio of *Penicillium* was often greater than 1 [14] (median = 1.002 in our study). Obviously, there is *Penicillium* contamination in an indoor environment when the I/O ratio is greater than 1, but contamination sources are rarely found.

For surface sampling, we collected data only from residential environments, but the most predominant fungal genera were also found on other indoor environmental surfaces, including those in green groceries, butcheries, houses, public restrooms, and laboratories [19]. Although commonly found surface fungi, including *Cladosporium*, *Penicillium*, and *Aspergillus* [19,20], are also frequently found in indoor air, fewer fungal genera were observed on the surface than in the air. Most surface sampling was only conducted for qualitative or semiquantitative analysis (no surface area available) for the identification of fungal genera. It would be difficult to directly compare the fungal densities on different surfaces. Compared to some dry regions with low rainfall, yeasts that appear in the environment may be related to the warm and humid climate in Taiwan. A previous study also demonstrated that yeasts may appear on relatively damp surfaces, such as bathroom sinks and bathtub drains [8].

This study investigated the four most important factors affecting indoor fungal concentrations, including temperature, RH, wind speed, and CO_2_ concentration. In general, the optimum temperature for different fungal taxa varies from 25 to 30 °C [21]. The results obtained for three airborne fungi are consistent with previous findings that airborne fungal concentrations are significantly positively correlated with temperature [17,22,23,24]. The growth of fungi as temperatures increase may also increase the bioavailability of airborne fungal allergens [24]. However, the concentration of surface fungi did not increase significantly with increasing surface temperature. We speculate that the conflicting results between air and surface samples may result from the smaller variation in surface temperature (coefficient of variation%; CV = 5.1%) than that of atmospheric temperature (CV = 6.7%). In addition, the World Health Organization (WHO) guidelines also indicate that common indoor temperatures may not be a limiting factor for fungal growth but may affect the growth rate [25]. In addition to the effect of temperature, we also found that RH, wind speed, and CO_2_ all have different correlations with airborne and surface fungi. RH is an important limiting factor for fungal growth, and most fungi may not grow below an RH of 75–80% [25,26]. CO_2_, an indicator of the ventilation rate, was negatively correlated with fungal concentrations, since air convection may change the fungal profile in air [27]. Conversely, CO_2_ may also promote the growth of specific fungi on the surface, such as *Aspergillus* spp. [28,29]. For the influence of wind speed, wind speed may accelerate changing the type and concentration of airborne fungi indoors, but it may also take away surface fungal spores and then reduce their concentration. The environmental factors investigated in this study may have different effects on air and surface fungi. However, the conflicting correlation results between airborne and surface samples may also result from the limitations of air and surface sampling technology. For example, the time for air sampling rarely exceeds 15 min and cannot represent a long-term fungal profile in the air [25]. The concentration and species profile of airborne fungi may vary rapidly [25]. Although surface sampling is considered less affected by temporal variation, it still cannot represent the airborne fungal concentration.

Taking into account the I/O ratios, higher fungal concentrations in outdoor air than in indoor air may reveal an exogenous source of indoor contamination. *Cladosporium* is a well-known outdoor genus with relatively constant concentrations year-round [7,8,16,19]. In addition, *Cladosporium*, *Aspergillus*, and *Fusarium* have been found to attach to the surfaces of plant leaves and may produce spores that float into the air [30]. *Geotrichum* (median I/O = 0.98) and *Verticillium* (median I/O = 0.78) not only exist in outdoor soil and plants but are also common plant pathogens [31,32]. Although *Penicillium* is also associated with soil and plants [16,30], it can still be found to originate in indoor sources since it can grow wherever organic material is available with very low water content [19]. For yeast, the median I/O ratio was greater than 1 (1.56), indicating that there may be an endogenous contamination source of indoor air. The indoor yeast concentration was negatively correlated with almost all molds but only positively correlated with *Cladosporium* and *Fusarium*. The correlation between yeast and *Fusarium* may be related to the water activity (Aw). Other molds, such as *Penicillium* and *Aspergillus*, are considered to have lower Aw values (0.85 or less) and therefore should be drought tolerant [33]. In contrast, both *Fusarium* and yeast grown at an Aw above 0.90 require higher amounts of available water [33,34].

In Table 4, it can be seen that the surface densities of individual fungi had no significant correlation with the corresponding airborne concentrations, except for those of *Geotrichum*. A previous study indicated that spores produced by specific molds cannot be easily aerosolized because of their sticky nature [4]. The dry spores produced by *Cladosporium*, *Penicillium*, and *Aspergillus* may have more chances to be propelled into the air. However, the recommendation by the previous study was based on the number of visible mold spots to predict air concentrations, not the mold density on the surface per area. Theoretically, the higher the concentration of surface fungi that can produce dry spores, the higher the air fungal concentration. A significant correlation was only observed for *Geotrichum*, which is a fast growing fungus with a powdery texture, whose dry spores can easily disperse through the air [35,36]. Researchers also found that *Geotrichum* can limit *Fusarium* growth because of available resource competition [35], and we did not find *Fusarium* on any surface in the current study. For other fungi, the low correlation between the surface and airborne fungal concentrations may be related to the fact that the naked eye can only observe mold spots of a certain size. In addition, some fungi growing at high Aw levels (such as yeast) tend to grow on relatively humid surfaces, and this kind of surface type and structure might have an impact on spore formation and therefore affect spore flux [4,37].

As shown in Table 5, we attempted to determine whether the ratio of the area of mold spots to the total house area would significantly contribute to a higher indoor fungal concentration. If there was a higher mold surface ratio (>0.00031) in the total area of an indoor environment, then there could be a higher airborne concentration of *Aspergillus* or *Geotrichum*. *Aspergillus* and *Geotrichum* may generate dry spores from surface mold spots, and these spores tend to proliferate from air to other organic compounds, such as food, paper, and dust, among others. Compared to *Geotrichum*, *Aspergillus* has received more attention because of its serious health impact [24]. *Aspergillus* species are commonly saprophytic fungi in nature, with small (2–3 μm) spores, and the concentration of *Aspergillus* spp. can significantly increase after a typhoon event [38]. We originally expected that *Cladosporium* and *Penicillium*, which have dry spores, might also contribute to higher indoor concentrations. However, the result was the same as that shown in Table 4. In terms of surface density and the surface area ratio, the airborne concentrations of *Cladosporium* and *Penicillium* contributed by surface molds may be quite limited. This phenomenon may be related to the small sizes of some mold spots of *Cladosporium* or *Penicillium,* which are too small to observe. Moreover, all our sampling families had children, so parents may have frequently cleaned up obviously visible mold spots.

Airborne *Verticillium* species are often found in agricultural areas, indoor environments, waste composting facilities, and even during Asian dust periods in Taiwan [39,40,41]. For *Verticillium*, the surface area ratio at level 2 (0–0.00031) may be related to the airborne concentration. However, this correlation was insignificant. Although we mainly explored the correlation between surface mold area and the concentration of airborne fungi, mold-infested surfaces may release more than spores into the air; a previous study also found that fungi on the surface of kitchens and showers may release volatile organic compounds into the air [42]. Thus, the appearance of surface mold affects human health and is worthy of further study. Conversely, we did not find a common fungus in Taiwan, *Alternaria* spp. A previous study indicated that *Alternaria* is related to agricultural plants [43]; thus, there may have been fewer opportunities to collect *Alternaria* as our sampling sites were mainly in residential areas. Another possible explanation is that we applied the culture assay for fungal quantification. The disadvantage of the culture method is that it may underestimate the viable fungal concentration, so that samples of *Alternaria* may be unavailable. Currently, several DNA-based methods, such as polymerase chain reaction (PCR), are available, but these methods may not distinguish dead or viable fungal cells without support from other technologies. Consequently, the microscopic method applied in this study maybe a complementary assay to culture-based methods for further identification of fungi.

Our study results recommend determining the surface area of visible mold spots, which may be important since a larger area of mold spot may be related to the airborne concentration of certain fungi. Quantifying areas by counting the number of pixels in a digital image has already been used in ecological and environmental studies [44,45]. Compared to manual quantification, the advantage of this method is that some irregular surface areas can be quickly quantified. However, there are some limitations of our study. First, some spores from the surface may not stick to the swab; therefore, the surface fungal concentration may be underestimated. Second, when observing the mold spot on site, if it is too small or white in color, it may be accidentally overlooked by the sampling personnel. Finally, young colonies and the colony margins of several *Aspergillus* and *Penicillium* species have a white color; therefore, the surface areas determined by digital image quantification can be underestimated.

## 4. Materials and Methods

### 4.1. Sampling Locations

This study involved several major cities in northern, middle, southern, and eastern Taiwan, including Taipei, Hsinchu, Yunlin, Chiayi, Tainan, Kaohsiung, and Taitung, with a total of 52 living environments being studied from June to September. The participating households all responded by questionnaire that there were visible mold spots on the walls of their houses.

### 4.2. Airborne Fungus Measurements

At each sampling site, fungal aerosol samples were collected in triplicate using an Andersen 1-STG impactor (Andersen Samplers, Inc., Atlanta, GA, USA). The pump and sampling apparatus were placed in the living room, bedroom, and outside entrance of each home. Among all the households included for indoor sampling, one had two master bedrooms and therefore had two bedroom samples. In another four households, because there was no obvious partition wall between the living room and the bedroom (studio apartment), only living room samples were collected. The sampling height was 1.2 m above the floor, within the breathing zone of individuals, at each location. The Andersen impactor was operated for 5 min with malt extract agar plates (MEAs) at a sampling flow rate of 28.3 L/min. All evaluated MEA plates collected from the field were placed in an incubator at 25 °C for 5 days. The fungal culture method recommended by the American Conference of Governmental Industrial Hygienists (ACGIH) was performed at room temperature (18 °C to 22 °C) [1]. In this study, we followed the standard method proposed by the Environmental Protection Administration in Taiwan (NIEA E401.15C) using MEA plates, which were incubated at 25 °C for 5 days. This incubation temperature has also been applied in previous studies [7,9,16]. Finally, the airborne fungal concentration presented in CFU/m^3^ was calculated based on the colony number, sampling time, and flow rate. For fungal species identification, all colony samples recovered from the MEA plates were subjected to lactophenol cotton blue staining and then identified under a microscope following the clinical laboratory handbook [46]. The reported genera included at least *Aspergillus*, *Penicillium*, *Cladosporium*, *Fusarium*, *Geotrichum*, *Verticillium*, and yeast. To increase the chance of collecting a variety of molds in the air, we collected three samples at each location. After cultivation, we determined the total counts of individual mold colonies recovered from the three agar plates and calculated their airborne concentrations separately.

### 4.3. Surface Sampling and Sampling Area Counting

**Figure 4:**
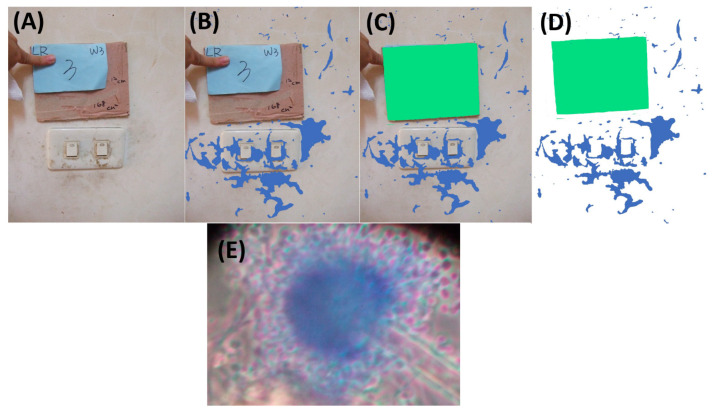
Field photo used to illustrate calculation of pixel numbers. (**A**) Raw picture of the wall surface. (**B**) Selection of each target spot as an individual layer (color blue). Subsequently, all layers of independent mold spots were combined, and the total pixels were counted using the software. (**C**) The reference paper was selected as another individual layer, and the pixels were counted using the software (color green). (**D**) Equation (1) was used to calculate the surface mold area. (**E**) The surface mold recovered from the MEA plate was subjected to lactophenol cotton blue staining and then identified as *Aspergillus* spp. by microscopic observation.

During the sampling period, visible molds on the surfaces of living room or bedroom walls were selected for swab sampling. Surface sampling was performed using a sterile water-moistened swab, and the surface was rubbed for 5 s. Subsequently, swabs were placed in a 2 mL tube containing 0.1% Tween 80 buffer and immediately transferred for culture. Aliquots (0.1 mL) of the liquid samples from the tubes were diluted 10- to 100-fold, plated on MEA plates, and then incubated for 5 days at 25 °C. Finally, the total colony counts of the target surfaces were calculated based on the dilution ratio, and the colony counts were adjusted by the surface areas to present the results in CFU/cm^2^. To quantify the sampling area, we calculated the target surface pixels in digital images, a method that has been applied to measure the area of a single leaf and hospital surfaces in ecological and environmental studies [44,47]. Briefly, we placed a reference paper beside the target surface and took a digital picture (Figure 4A). While taking the picture, the angle between the wall surface and camera was aimed vertically at the target center. Therefore, we obtained a picture including the reference paper and surface of interest and then directly calculated the pixel numbers in Photoshop. The procedure to calculate the pixel numbers in Photoshop is demonstrated in Figure 4.

**Figure 5:**
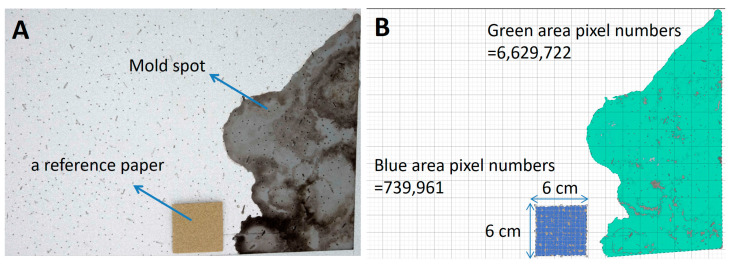
Raw (**A**) and processed (**B**) pictures were used to calculate the surface area of visible mold spots on wall surfaces. The number of pixels in the reference paper (6 × 6 cm^2^) and the surface of the mold were calculated using the processed photographs (**B**). The surface mold spot area in picture B is approximately 323 cm^2^, based on Equation (1) and described in the methods section.

The surface area of interest was calculated via the following Equation:

(1) $$\frac{A_{\mathrm{surface}}}{A_{\mathrm{reference}\;\mathrm{paper}}}=\frac{N_{\mathrm{surface}}}{N_{\mathrm{reference}\;\mathrm{paper}}}$$

where A_surface_ and A_reference paper_ are the areas of the target surface and reference paper, respectively, and N_surface_ and N_reference paper_ are the pixel numbers of the target surface and reference paper counted in Photoshop within the same picture, respectively (Figure 5).

### 4.4. Other Environmental Parameter Measurements

The temperature, atmospheric relative humidity (RH), and concentration of carbon dioxide (CO_2_) were recorded using a KD AirBoxx real-time instrument (KD Engineering, Inc., Blaine, WA, USA) in all sampling locations. In addition to atmospheric RH, we also measured the surface temperature and moisture content of the target surface where visible molds appeared using an HP23-AW handheld instrument (Rotronic AG, Bassersdorf, Swiss). Conversely, the wind speed was measured using an air velocity meter (TSI 9535, Shoreview, MN, USA)

### 4.5. Statistical Analysis

Differences in airborne fungal concentrations between different sampling locations were determined by the Wilcoxon signed rank test. For the individual pairwise correlations between environmental parameters and airborne/surface fungal concentrations, Spearman’s rank correlation model was constructed. Finally, to further explore the correlation between the mold surface areas and their concentrations in the air, a multiple linear regression model was constructed. A natural logarithm transformation (ln-transformation) of airborne fungal concentration was performed before conducting the analyses. The corresponding regression coefficients were back-transformed (100 × (e^β^ − 1)) to obtain the percent changes. The limit of detection (LOD) was 2.36 CFU/m^3^ for airborne fungal samples and 1.33 CFU/cm^2^ for surface samples. The airborne or surface samples with individual fungal concentrations below the LOD were assigned a value of one-third of the LOD for the statistical analyses.

## 5. Conclusions

Rapid monitoring of fungi is important for evaluating the related health risks and maintaining good indoor air quality. This study demonstrated that the indoor air concentration of individual fungi is significantly correlated with the outdoor air concentration. Regarding the contribution of surface fungi to indoor air concentrations, the density of individual fungi on the surface had no significant correlation with indoor air concentrations, except for *Geotrichum*. However, for some specific genera, including *Aspergillus* and *Geotrichum*, the surface area of visible mold spots was significantly related to their airborne concentration indoors. These results indicate the need to focus attention on visible mold spots on surfaces, which may contain fungal spores of certain allergens, such as *Aspergillus*, with the ability to further disperse from the surface into the air through environmental forces. In addition, this study also provides a convenient and rapid method for quantifying the area of surface mold spots. This method may be applied in future environmental monitoring or epidemiological studies.

## Figures and Tables

**Table 1:** Spearman’s rank correlation coefficient with environmental parameters and fungal concentrations in the indoor air.

	Temperature ^#^		Atmospheric RH ^#^		Wind Speed		CO_2_	
	r	*p*	r	*p*	r	*p*	r	*p*
*Cladosporium* spp.	0.195 *	0.048	−0.149	0.133	0.094	0.345	−0.200 *	0.042
*Penicillium* spp.	−0.064	0.524	0.397 **	<0.0001	−0.020	0.842	0.023	0.819
*Aspergillus* spp.	0.063	0.525	0.118	0.235	−0.156	0.116	−0.279 **	0.004
*Fusarium* spp.	0.205 *	0.038	−0.274 **	0.005	−0.001	0.992	−0.419 **	<0.0001
*Geotrichum* spp.	0.028	0.783	0.062	0.532	−0.054	0.586	0.010	0.922
Yeast	−0.016	0.874	−0.157	0.114	0.070	0.485	0.038	0.702
*Verticillium* spp.	0.247 *	0.012	−0.269 **	0.006	0.237 *	0.016	−0.246 *	0.012
Total fungi	0.144	0.148	−0.026	0.798	0.156	0.115	−0.273 **	0.005

N = 103, * *p* < 0.05, ** *p* < 0.01, ^#^ Temperature = Atmospheric Temperature, ^#^ RH = Relative humidity.

**Table 2:** Spearman’s rank correlation coefficient with environmental parameters and fungal concentrations on the surface.

	Surface Tem ^#^		Surface RH ^#^		Wind Speed		CO_2_	
	r	*p*	r	*p*	r	*p*	r	*p*
*Cladosporium* spp.	0.1571	0.113	0.0344	0.730	−0.1073	0.281	−0.0050	0.960
*Penicillium* spp.	0.1573	0.112	0.1802	0.069	−0.0636	0.523	−0.0130	0.896
*Aspergillus* spp.	0.0807	0.418	0.0435	0.663	−0.0915	0.358	0.0610	0.541
*Geotrichum* spp.	0.1050	0.291	0.2325 *	0.018	−0.0275	0.783	0.0117	0.907
Yeast	−0.073	0.462	0.1467	0.091	−0.143	0.149	0.335 **	0.001
*Verticillium* spp.	0.0355	0.722	0.2425 *	0.014	−0.0729	0.464	−0.0088	0.930
Total fungi	0.0934	0.348	0.1594	0.108	−0.2302 *	0.019	0.3021 **	0.002

N = 103, * *p* < 0.05, ** *p* < 0.01, ^#^ Surface Tem = Surface Temperature, ^#^ RH = Relative humidity.

**Table 3:** Spearman’s rank correlation coefficient with airborne fungal concentrations in the indoor and outdoor air.

	ID ^#^ cla	ID pen	ID asp	ID fus	ID geo	ID Yeast	ID ver	ID Total	OD ^#^ cla	OD pen	OD asp	OD fus	OD geo	OD Yeast	OD ver	OD Total
ID cla	1.000															
ID pen	−0.231 *	1.000														
ID asp	0.235 *	0.110	1.000													
ID fus	0.397 **	−0.247 *	0.242 *	1.000												
ID geo	−0.264 **	0.141	−0.160	−0.061	1.000											
ID yeast	0.043	−0.411 **	−0.085	0.077	−0.136	1.000										
ID ver	0.294 **	−0.115	−0.115	0.265 **	−0.083	−0.137	1.000									
ID total	0.737 **	−0.096	0.186	0.298 **	−0.260 **	0.312 **	0.242 *	1.000								
OD cla	0.716 **	−0.258 **	0.275 **	0.513 **	−0.207 *	−0.007	0.301 **	0.451 **	1.000							
OD pen	−0.328 **	0.810 **	0.093	−0.117	0.314 **	−0.456 **	−0.134	−0.291 **	−0.151	1.000						
OD asp	0.340 **	−0.056	0.484 **	0.289 **	−0.250 *	−0.144	0.105	0.173	0.469 **	−0.002	1.000					
OD fus	0.475 **	−0.205 *	0.147	0.585 **	−0.082	−0.040	0.222 *	0.221 *	0.640 **	−0.028	0.272 **	1.000				
OD geo	−0.317 **	0.140	−0.090	−0.054	0.793 **	−0.024	−0.073	−0.223 *	−0.198 *	0.256 **	−0.289 **	−0.126	1.000			
OD yeast	0.169	−0.466 **	−0.091	0.113	−0.173	0.697 **	−0.071	0.320 **	0.219 *	−0.467 **	0.049	0.149	−0.214 *	1.000		
OD ver	0.294 **	−0.116	−0.114	0.265 **	−0.083	−0.137	1.000 **	0.242 *	0.301 **	−0.134	0.105	0.222 *	−0.073	−0.071	1.000	
OD total	0.670 **	−0.210 *	0.321 **	0.525 **	−0.112	0.131	0.231 *	0.583 **	0.856 **	−0.149	0.504 **	0.571 **	−0.124	0.430 **	0.231 *	1.000

N = 103, * *p* < 0.05, ** *p* < 0.01, ^#^ ID = Indoor air; OD ^#^ = Outdoor, cla = *Cladosporium*; pen = *Penicillium*; asp = *Aspergillus*; fus = *Fusarium*; geo = *Geotrichum*; ver = *Verticillium*; total = total fungi.

**Table 4:** Spearman’s rank correlation coefficient with the fungal concentrations in the indoor air and on the surface.

	ID ^#^ cla	ID pen	ID asp	ID geo	ID yeast	ID ver	ID Total	SF ^#^ cla	SF pen	SF asp	SF geo	SF Yeast	SF ver	SF Total
ID cla	1.000													
ID pen	−0.231 *	1.000												
ID asp	0.235 *	0.110	1.000											
ID geo	−0.264 **	0.141	−0.160	1.000										
ID yeast	0.043	−0.411 **	−0.085	−0.136	1.000									
ID ver	0.294 **	−0.115	−0.115	−0.083	−0.137	1.000								
ID total	0.737 **	−0.096	0.186	−0.260 **	0.312 **	0.242 *	1.000							
SF cla	0.063	0.109	0.295 **	0.181	0.002	−0.010	0.197 *	1.000						
SF pen	−0.088	0.185	0.178	0.322 **	0.022	0.288 **	0.022	0.381 **	1.000					
SF asp	−0.013	−0.087	0.081	0.184	−0.043	0.137	−0.047	0.300 **	0.424 **	1.000				
SF geo	−0.303 **	0.282 **	0.095	0.420 **	0.014	−0.054	−0.054	0.504 **	0.704 **	0.389 **	1.000			
SF yeast	0.061	0.003	−0.057	0.101	0.051	0.009	0.052	−0.176	0.053	−0.154	−0.143	1.000		
SF ver	−0.198 *	0.271 **	0.105	0.361 **	0.065	−0.035	−0.029	0.389 **	0.563 **	0.402 **	0.655 **	−0.089	1.000	
SF total	0.108	0.032	0.131	0.269 **	0.078	0.020	0.176	0.537 **	0.401 **	0.291 **	0.312 **	0.623 **	0.303 **	1.000

N = 103, * *p* < 0.05, ** *p* < 0.01, ^#^ ID = Indoor air; SF ^#^ = Surface, cla = *Cladosporium*; pen = *Penicillium*; asp = *Aspergillus*; geo = *Geotrichum*; ver = *Verticillium*; total = total fungi.

**Table 5:** Association among the airborne fungal concentration, fungal surface area ratio and environmental parameters determined by multiple linear regression model.

	N	ID ^#^ *cla*	ID *pen*	ID *asp*	ID *geo*	ID *Yeast*	ID *ver*	ID *Total*
		Percent Changes (β ± SE)						
Surface area ratio								
Level 1:0	56	Reference	Reference	Reference	Reference	Reference	Reference	Reference
Level 2: ≤Median (>0–0.00031)	24	−8.11 ± 37.14	−55.42 ± 58.10	61.0 ± 34.94	23.63 ± 27.49	10.86 ± 76.8	39.8 ± 22.92	7.5 ± 22.73
Level 3: >Median (0.00031–1)	23	33.02 ± 38.02	112.19 ± 59.57	138.8 ± 35.7 **	85.5 ± 28.12 *	1.39 ± 78.88	2.53 ± 23.44	39.03 ± 23.24
Atmospheric Temperature	103	11.68 ± 7.22	5.62 ± 10.63	4.37 ± 6.83	1.24 ± 5.5	−4.42 ± 13.40	4.61 ± 4.66	10.33 ± 4.62 *
Atmospheric humidity	103	−0.33 ± 1.46	7.76 ± 2.13 **	1.31 ± 1.39	0.19 ± 1.12	−3.81 ± 2.65	−1.46 ± 0.95	1.21 ± 0.94
CO_2_	103	−0.05 ± 0.07	−0.04 ± 0.10	−0.23 ± 0.06 **	−0.03 ± 0.05	0.03 ± 0.12	−0.08 ± 0.04	−0.12 ± 0.04 **

* *p* < 0.05 ** *p* < 0.01, ^#^ ID = Indoor air, cla = *Cladosporium*; pen = *Penicillium*; asp = *Aspergillus*; geo = *Geotrichum*; ver = *Verticillium*; total = total fungi.

## Data Availability

The original measurement data are available in the electronic supplement. Samples with the same location ID number were collected in the same household. ID#B indicates that sampling was conducted in the bedroom, and ID#L represents the living room samples.

## Supplementary Materials

The following are available online at https://www.mdpi.com/article/10.3390/pathogens10081032/s1.
