# Supplementary material for: Contribution of Visible Surface Mold to Airborne Fungal Concentration as Assessed by Digital Image Quantification
Source: Pathogens. 2021 Aug 15;10(8):1032. doi: 10.3390/pathogens10081032 (PMC8400061; doi:10.3390/pathogens10081032)
Supplement: Supplementary file 1 [file pathogens-10-01032-s001.zip › pathogens-1287550-supplementary.pdf]

| LOCATION_ID | DATE | AREA_RATIO | INDOOR AIR | ID_CLA2 | ID_PEN2 | ID_ASP2 | ID_FUSARIU | ID_GEO2 | ID_YEAST2 | ID_VER2 | ID_TOTAL | OUTDOOR AIR | OD_CLA | OD_PEN | OD_ASP | OD_FUSARIU | OD_GEO | OD_YEAST | OD_VER | OD_TOTAL | SURFACE | SURFACE_CL | SURFACE_PE | SURFACE_AS | SURFACE_GE | SURFACE_YE | SURFACE_VE | SURFACE_TO | ENVIRONMENT | ID_TEM | ID_RH | WIND_SPEED | CO2 | SURFACE_TEM | SURFACE_RH |
|---|---|---|---|---|---|---|---|---|---|---|---|---|---|---|---|---|---|---|---|---|---|---|---|---|---|---|---|---|---|---|---|---|---|---|---|
| 13B | 6/29 | 0.002400 | | 33.63 | 595.01 | 4.80 | 0.79 | 0.79 | 0.79 | 0.79 | 647.82 | | 36.93 | 76.22 | 7.40 | 0.79 | 0.79 | 2.42 | 0.79 | 171.97 | | 2.22 | 0.440 | 0.440 | 6.670 | 0.440 | 0.440 | 8.890 | | 31.5 | 73.4 | 0.26 | 700 | 32.42 | 80.30 |
| 13B | 6/29 | 0.000170 | | 88.11 | 173.94 | 7.31 | 0.79 | 0.79 | 9.89 | 0.79 | 308.60 | | 36.93 | 76.22 | 7.40 | 0.79 | 0.79 | 2.42 | 0.79 | 171.97 | | 2.67 | 1.333 | 0.440 | 2.667 | 0.440 | 0.440 | 6.667 | | 30.6 | 73.5 | 0.20 | 650 | 33.20 | 72.40 |
| 13L | 6/29 | 0.000000 | | 9.59 | 190.48 | 0.79 | 0.79 | 0.79 | 0.79 | 0.79 | 221.44 | | 36.93 | 76.22 | 7.40 | 0.79 | 0.79 | 2.42 | 0.79 | 171.97 | | 0.44 | 0.440 | 0.440 | 0.440 | 0.440 | 0.440 | 0.440 | | 30.9 | 72.3 | 0.35 | 650 | 32.87 | 73.93 |
| 14B | 6/29 | 0.000710 | | 140.79 | 59.25 | 4.92 | 0.79 | 0.79 | 7.45 | 0.79 | 252.06 | | 87.76 | 43.88 | 19.50 | 0.79 | 0.79 | 12.19 | 0.79 | 209.66 | | 3.33 | 0.440 | 0.440 | 0.440 | 0.440 | 0.440 | 3.330 | | 26.0 | 63.3 | 0.16 | 600 | 27.82 | 62.35 |
| 14L | 6/29 | 0.005910 | | 71.49 | 39.62 | 4.85 | 0.79 | 22.24 | 24.74 | 0.79 | 200.24 | | 87.76 | 43.88 | 19.50 | 0.79 | 0.79 | 12.19 | 0.79 | 209.66 | | 13.33 | 26.667 | 0.440 | 96.667 | 0.440 | 0.440 | 136.667 | | 28.8 | 73.4 | 0.10 | 500 | 31.40 | 65.18 |
| 16B | 6/29 | 0.000000 | | 43.97 | 39.19 | 0.79 | 0.79 | 0.79 | 17.06 | 0.79 | 127.21 | | 16.49 | 35.34 | 7.07 | 0.79 | 0.79 | 2.36 | 0.79 | 103.65 | | 0.44 | 0.440 | 0.440 | 0.440 | 0.440 | 0.440 | 0.440 | | 31.7 | 59.0 | 0.18 | 800 | 32.10 | 63.76 |
| 16L | 6/29 | 0.000000 | | 45.89 | 53.28 | 7.22 | 0.79 | 0.79 | 2.43 | 0.79 | 183.75 | | 16.49 | 35.34 | 7.07 | 0.79 | 0.79 | 2.36 | 0.79 | 103.65 | | 0.44 | 0.440 | 0.440 | 0.440 | 0.440 | 0.440 | 0.440 | | 33.6 | 60.3 | 0.20 | 550 | 31.82 | 72.04 |
| 15B | 6/30 | 0.000000 | | 26.53 | 370.15 | 2.40 | 0.79 | 0.79 | 48.40 | 0.79 | 452.30 | | 114.25 | 96.85 | 0.79 | 0.79 | 2.46 | 0.79 | 0.79 | 228.50 | | 0.44 | 0.440 | 0.440 | 0.440 | 0.440 | 0.440 | 0.440 | | 27.3 | 66.2 | 0.12 | 600 | 31.17 | 65.20 |
| 15L | 6/30 | 0.002850 | | 48.10 | 0.79 | 33.18 | 0.79 | 19.34 | 19.53 | 0.79 | 122.50 | | 36.51 | 24.15 | 2.36 | 0.79 | 23.75 | 0.79 | 0.79 | 96.58 | | 16.67 | 20.000 | 933.333 | 66.667 | 0.440 | 0.440 | 1036.667 | | 29.7 | 61.6 | 0.07 | 550 | 31.05 | 70.70 |
| 22B | 7/8 | 0.005160 | | 307.36 | 0.79 | 23.29 | 20.64 | 0.79 | 462.25 | 0.79 | 857.48 | | 351.16 | 0.79 | 43.58 | 0.79 | 0.79 | 341.47 | 0.79 | 751.47 | | 7923.56 | 0.440 | 0.440 | 0.440 | 0.440 | 0.440 | 7923.560 | | 34.4 | 58.4 | 0.12 | 700 | 34.59 | 63.79 |
| 22L | 7/8 | 0.001440 | | 465.11 | 13.15 | 28.78 | 5.17 | 0.79 | 514.42 | 0.79 | 1031.80 | | 351.16 | 0.79 | 43.58 | 5.15 | 0.79 | 341.47 | 0.79 | 751.47 | | 6224.60 | 0.440 | 0.440 | 0.440 | 0.440 | 0.440 | 6224.600 | | 33.5 | 56.8 | 0.01 | 800 | 35.08 | 59.07 |
| 29B | 7/8 | 0.000000 | | 51.83 | 9.42 | 14.13 | 2.36 | 0.79 | 9.42 | 0.79 | 94.23 | | 137.14 | 0.79 | 62.44 | 0.79 | 0.79 | 24.21 | 0.79 | 226.15 | | 0.44 | 0.440 | 0.440 | 0.440 | 0.440 | 0.440 | 0.440 | | 29.2 | 60.1 | 0.16 | 650 | 31.08 | 61.98 |
| 29L | 7/8 | 0.000000 | | 62.21 | 2.36 | 7.15 | 21.99 | 0.79 | 28.79 | 0.79 | 122.50 | | 137.14 | 0.79 | 62.44 | 0.79 | 0.79 | 24.21 | 0.79 | 226.15 | | 0.44 | 0.440 | 0.440 | 0.440 | 0.440 | 0.440 | 0.440 | | 27.8 | 66.7 | 0.19 | 500 | 31.40 | 60.43 |
| 50L | 7/8 | 0.000000 | | 1139.97 | 11.88 | 9.51 | 0.79 | 0.79 | 0.79 | 0.79 | 1161.37 | | 253.03 | 0.79 | 43.39 | 5.15 | 0.79 | 0.79 | 0.79 | 313.31 | | 0.44 | 0.440 | 0.440 | 0.440 | 0.440 | 0.440 | 0.440 | | 31.3 | 65.5 | 0.05 | 450 | 32.18 | 72.38 |
| 51L | 7/8 | 0.000240 | | 225.44 | 0.79 | 7.20 | 31.20 | 0.79 | 0.79 | 0.79 | 263.84 | | 446.70 | 0.79 | 18.98 | 86.25 | 0.79 | 45.24 | 0.79 | 614.84 | | 0.44 | 0.440 | 15.560 | 0.440 | 0.440 | 0.440 | 15.560 | | 30.7 | 67.2 | 0.00 | 650 | 31.72 | 73.84 |
| 52L | 7/8 | 0.002780 | | 79.42 | 67.19 | 12.36 | 0.79 | 0.79 | 34.21 | 0.79 | 193.17 | | 53.96 | 105.52 | 9.86 | 2.44 | 0.79 | 4.90 | 0.79 | 176.68 | | 58.33 | 0.440 | 0.440 | 0.440 | 0.440 | 0.440 | 58.330 | | 33.1 | 63.9 | 0.13 | 581 | 32.90 | 69.77 |
| 37B | 7/20 | 0.000000 | | 228.01 | 4.84 | 4.71 | 11.96 | 0.79 | 16.68 | 0.79 | 270.91 | | 389.74 | 4.84 | 7.25 | 79.28 | 0.79 | 0.79 | 0.79 | 447.59 | | 0.44 | 0.440 | 0.440 | 0.440 | 0.440 | 0.440 | 0.440 | | 29.9 | 56.0 | 0.10 | 550 | 31.60 | 56.90 |
| 37L | 7/20 | 0.000000 | | 325.70 | 0.79 | 14.49 | 50.77 | 0.79 | 0.79 | 0.79 | 393.40 | | 389.74 | 4.84 | 7.25 | 36.33 | 0.79 | 0.79 | 0.79 | 447.59 | | 0.44 | 0.440 | 0.440 | 0.440 | 0.440 | 0.440 | 0.440 | | 29.9 | 70.8 | 0.58 | 450 | 31.20 | 62.78 |
| 40B | 7/20 | 0.000070 | | 169.05 | 0.79 | 23.05 | 79.42 | 0.79 | 574.17 | 0.79 | 845.70 | | 161.00 | 0.79 | 20.21 | 79.28 | 0.79 | 429.72 | 0.79 | 690.22 | | 6.67 | 0.440 | 0.440 | 0.440 | 0.440 | 0.440 | 6.667 | | 31.7 | 59.7 | 0.17 | 500 | 32.10 | 63.20 |
| 40L | 7/20 | 0.000000 | | 139.56 | 0.79 | 4.96 | 52.29 | 0.79 | 410.96 | 0.79 | 607.77 | | 161.00 | 0.79 | 20.21 | 0.79 | 0.79 | 429.72 | 0.79 | 690.22 | | 0.44 | 0.440 | 0.440 | 0.440 | 0.440 | 0.440 | 0.440 | | 28.8 | 58.0 | 0.06 | 550 | 31.90 | 60.20 |
| 41B | 7/20 | 0.000430 | | 395.65 | 16.58 | 9.45 | 0.79 | 0.79 | 0.79 | 0.79 | 421.67 | | 253.03 | 0.79 | 43.39 | 0.79 | 0.79 | 0.79 | 0.79 | 313.31 | | 130.00 | 0.440 | 0.440 | 0.440 | 0.440 | 0.440 | 130.000 | | 31.3 | 65.5 | 0.05 | 450 | 32.18 | 72.38 |
| 41L | 7/20 | 0.000000 | | 251.50 | 0.79 | 19.19 | 0.79 | 0.79 | 0.79 | 0.79 | 285.04 | | 253.03 | 0.79 | 43.39 | 0.79 | 0.79 | 0.79 | 0.79 | 313.31 | | 0.44 | 0.440 | 0.440 | 0.440 | 0.440 | 0.440 | 0.440 | | 31.0 | 67.8 | 1.45 | 400 | 31.88 | 71.16 |
| 01B | 7/21 | 0.001070 | | 195.59 | 0.79 | 4.93 | 44.50 | 0.79 | 204.92 | 0.79 | 449.94 | | 443.09 | 0.79 | 22.29 | 112.02 | 0.79 | 27.41 | 0.79 | 614.84 | | 110.00 | 0.440 | 0.440 | 0.440 | 0.440 | 0.440 | 110.000 | | 26.5 | 58.2 | 0.06 | 500 | 32.27 | 54.17 |
| 01L | 7/21 | 0.000000 | | 442.41 | 0.79 | 17.56 | 92.93 | 0.79 | 75.75 | 0.79 | 633.69 | | 443.09 | 0.79 | 22.29 | 112.02 | 0.79 | 27.41 | 0.79 | 614.84 | | 0.44 | 0.440 | 0.440 | 0.440 | 0.440 | 0.440 | 0.440 | | 33.1 | 60.8 | 0.24 | 400 | 29.13 | 79.13 |
| 43B | 7/21 | 0.000000 | | 181.11 | 0.79 | 10.93 | 15.22 | 0.79 | 34.30 | 0.79 | 247.35 | | 446.70 | 0.79 | 18.98 | 36.33 | 0.79 | 45.24 | 0.79 | 614.84 | | 0.44 | 0.440 | 0.440 | 0.440 | 0.440 | 0.440 | 0.440 | | 28.7 | 60.8 | 0.12 | 550 | 32.30 | 59.78 |
| 43L | 7/21 | 0.000000 | | 322.36 | 0.79 | 17.20 | 46.94 | 0.79 | 37.07 | 0.79 | 433.45 | | 446.70 | 0.79 | 18.98 | 86.25 | 0.79 | 45.24 | 0.79 | 614.84 | | 0.44 | 0.440 | 0.440 | 0.440 | 0.440 | 0.440 | 0.440 | | 31.7 | 65.6 | 0.30 | 550 | 31.26 | 60.04 |
| 47B | 7/21 | 0.000140 | | 301.53 | 0.79 | 14.95 | 117.28 | 0.79 | 72.72 | 0.79 | 506.48 | | 248.04 | 0.79 | 15.12 | 106.22 | 0.79 | 233.68 | 0.79 | 603.06 | | 0.44 | 0.440 | 0.440 | 0.440 | 20.000 | 0.440 | 20.000 | | 36.2 | 52.3 | 0.07 | 450 | 36.70 | 57.78 |
| 47L | 7/21 | 0.000000 | | 320.05 | 0.79 | 12.67 | 90.90 | 0.79 | 186.50 | 0.79 | 610.13 | | 248.04 | 0.79 | 15.12 | 106.22 | 0.79 | 233.68 | 0.79 | 603.06 | | 0.44 | 0.440 | 0.440 | 0.440 | 0.440 | 0.440 | 0.440 | | 35.3 | 54.6 | 0.33 | 450 | 36.80 | 57.24 |
| 38B | 7/22 | 0.000310 | | 442.76 | 4.77 | 0.79 | 0.79 | 7.12 | 0.79 | 0.79 | 454.65 | | 968.04 | 59.42 | 0.79 | 90.18 | 0.79 | 481.85 | 0.79 | 1710.25 | | 143.33 | 0.440 | 0.440 | 0.440 | 0.440 | 0.440 | 143.330 | | 28.8 | 54.7 | 0.29 | 550 | 30.70 | 53.29 |
| 38L | 7/22 | 0.000000 | | 649.46 | 19.28 | 0.79 | 120.63 | 0.79 | 616.99 | 0.79 | 1406.36 | | 968.04 | 59.42 | 0.79 | 200.93 | 0.79 | 481.85 | 0.79 | 1710.25 | | 0.44 | 0.440 | 0.440 | 0.440 | 0.440 | 0.440 | 0.440 | | 30.8 | 55.8 | 0.10 | 550 | 31.80 | 47.34 |
| 39L | 7/22 | 0.000190 | | 824.05 | 0.79 | 4.99 | 77.27 | 0.79 | 44.74 | 0.79 | 963.49 | | 823.47 | 0.79 | 4.98 | 90.18 | 0.79 | 62.75 | 0.79 | 996.47 | | 280.00 | 0.440 | 0.440 | 0.440 | 0.440 | 0.440 | 280.000 | | 30.8 | 67.6 | 0.42 | 450 | 34.94 | 61.64 |
| 05B | 7/25 | 0.000380 | | 85.27 | 99.74 | 26.79 | 7.28 | 0.79 | 0.79 | 0.79 | 219.08 | | 273.85 | 111.56 | 70.37 | 22.00 | 0.79 | 7.41 | 0.79 | 475.85 | | 0.44 | 5.000 | 0.440 | 0.440 | 15.000 | 0.440 | 20.000 | | 30.9 | 81.0 | 0.00 | 1041 | 31.73 | 80.72 |
| 05L | 7/25 | 0.000030 | | 185.73 | 108.97 | 29.66 | 14.85 | 0.79 | 0.79 | 0.79 | 339.22 | | 273.85 | 111.56 | 70.37 | 12.67 | 0.79 | 7.41 | 0.79 | 475.85 | | 1.67 | 0.440 | 0.440 | 0.440 | 0.440 | 0.440 | 1.667 | | 28.7 | 82.9 | 0.00 | 547 | 29.77 | 85.47 |
| 06B | 7/26 | 0.000250 | | 118.48 | 111.04 | 12.31 | 0.79 | 0.79 | 17.29 | 0.79 | 259.13 | | 119.31 | 82.71 | 2.43 | 12.67 | 0.79 | 9.78 | 0.79 | 219.08 | | 0.44 | 0.440 | 0.440 | 0.440 | 1.670 | 0.440 | 1.670 | | 29.9 | 75.6 | 0.36 | 500 | 32.50 | 72.03 |
| 06L | 7/26 | 0.000000 | | 132.11 | 105.46 | 7.42 | 0.79 | 0.79 | 0.79 | 0.79 | 244.99 | | 119.31 | 82.71 | 2.43 | 4.86 | 0.79 | 9.78 | 0.79 | 219.08 | | 0.44 | 0.440 | 0.440 | 0.440 | 0.440 | 0.440 | 0.440 | | 31.6 | 71.4 | 0.05 | 504 | 31.80 | 72.97 |
| 07B | 7/26 | 0.000000 | | 79.42 | 67.19 | 12.36 | 0.79 | 0.79 | 34.21 | 0.79 | 193.17 | | 53.96 | 105.52 | 9.86 | 4.86 | 0.79 | 4.90 | 0.79 | 176.68 | | 0.44 | 0.440 | 0.440 | 0.440 | 0.440 | 0.440 | 0.440 | | 33.1 | 63.9 | 0.13 | 581 | 33.07 | 66.83 |
| 07L | 7/26 | 0.000000 | | 67.62 | 99.15 | 4.80 | 7.31 | 0.79 | 7.22 | 0.79 | 186.10 | | 53.96 | 105.52 | 9.86 | 2.44 | 0.79 | 4.90 | 0.79 | 176.68 | | 0.44 | 0.440 | 0.440 | 0.440 | 0.440 | 0.440 | 0.440 | | 32.7 | 64.7 | 0.25 | 500 | 33.20 | 64.85 |
| 32B | 7/27 | 0.000000 | | 635.00 | 14.65 | 2.42 | 65.75 | 0.79 | 0.79 | 17.16 | 734.98 | | 452.30 | 9.75 | 12.17 | 40.34 | 0.79 | 0.79 | 44.18 | 577.15 | | 0.44 | 0.440 | 0.440 | 0.440 | 0.440 | 0.440 | 0.440 | | 31.5 | 56.2 | 0.28 | 450 | 32.62 | 60.04 |
| 32L | 7/27 | 0.000000 | | 548.23 | 24.40 | 2.47 | 38.77 | 0.79 | 0.79 | 24.53 | 638.40 | | 452.30 | 9.75 | 12.17 | 58.75 | 0.79 | 0.79 | 44.18 | 577.15 | | 0.44 | 0.440 | 0.440 | 0.440 | 0.440 | 0.440 | 0.440 | | 32.3 | 53.8 | 0.30 | 450 | 32.80 | 63.54 |
| 35B | 7/27 | 0.000040 | | 185.94 | 7.34 | 7.35 | 12.25 | 0.79 | 39.18 | 0.79 | 252.06 | | 351.18 | 12.13 | 9.69 | 200.93 | 0.79 | 29.14 | 0.79 | 426.38 | | 10.00 | 0.440 | 0.440 | 0.440 | 56.670 | 0.440 | 66.670 | | 31.2 | 57.9 | 0.06 | 700 | 31.32 | 64.76 |
| 35L | 7/27 | 0.000000 | | 253.40 | 12.04 | 4.76 | 12.01 | 0.79 | 28.74 | 0.79 | 310.95 | | 351.18 | 12.13 | 9.69 | 24.24 | 0.79 | 29.14 | 0.79 | 426.38 | | 0.44 | 0.440 | 0.440 | 0.440 | 0.440 | 0.440 | 0.440 | | 32.8 | 56.7 | 0.33 | 500 | 32.24 | 66.08 |
| 36B | 7/27 | 0.000170 | | 465.95 | 0.79 | 7.68 | 48.65 | 0.79 | 15.47 | 143.06 | 680.80 | | 716.14 | 0.79 | 10.10 | 24.24 | 0.79 | 20.13 | 141.44 | 928.15 | | 0.44 | 2366.670 | 0.440 | 0.440 | 900.000 | 0.440 | 3266.670 | | 32.4 | 57.8 | 0.20 | 500 | 33.65 | 63.64 |
| 36L | 7/27 | 0.000030 | | 565.63 | 0.79 | 2.58 | 18.07 | 0.79 | 10.32 | 152.52 | 749.12 | | 716.14 | 0.79 | 10.10 | 40.34 | 0.79 | 20.13 | 141.44 | 928.15 | | 0.56 | 26.667 | 26.667 | 0.440 | 0.440 | 0.440 | 26.667 | | 32.8 | 55.1 | 0.67 | 450 | 33.00 | 60.90 |
| 49B | 7/27 | 0.000000 | | 189.86 | 2.40 | 7.16 | 0.79 | 0.79 | 19.22 | 0.79 | 240.28 | | 373.07 | 9.89 | 9.85 | 41.53 | 0.79 | 32.09 | 0.79 | 466.43 | | 0.44 | 0.440 | 0.440 | 0.440 | 0.440 | 0.440 | 0.440 | | 27.4 | 48.5 | 0.20 | 850 | 29.53 | 49.25 |
| 03B | 7/28 | 0.000000 | | 45.29 | 0.79 | 7.17 | 21.63 | 0.79 | 84.16 | 0.79 | 136.63 | | 331.09 | 4.89 | 4.71 | 2.51 | 0.79 | 28.94 | 0.79 | 376.91 | | 0.44 | 0.440 | 0.440 | 0.440 | 0.440 | 0.440 | 0.440 | | 29.2 | 48.2 | 0.08 | 525 | 30.50 | 53.16 |
| 03L | 7/28 | 0.000000 | | 256.88 | 9.73 | 12.09 | 16.97 | 0.79 | 41.20 | 0.79 | 336.87 | | 373.07 | 9.89 | 9.85 | 41.53 | 0.79 | 32.09 | 0.79 | 466.43 | | 0.44 | 0.440 | 0.440 | 0.440 | 0.440 | 0.440 | 0.440 | | 30.3 | 57.0 | 0.12 | 500 | 30.64 | 64.36 |
| 31B | 7/28 | 0.000020 | | 21.20 | 0.79 | 9.42 | 0.79 | 0.79 | 9.42 | 0.79 | 42.40 | | 101.18 | 0.79 | 0.79 | 2.40 | 0.79 | 266.16 | 0.79 | 369.85 | | 0.44 | 0.440 | 0.440 | 0.440 | 1183.330 | 0.440 | 1183.330 | | 33.0 | 48.4 | 0.12 | 650 | 34.26 | 51.92 |
| 31L | 7/28 | 0.000000 | | 149.01 | 0.79 | 7.28 | 17.15 | 0.79 | 585.00 | 0.79 | 760.90 | | 101.18 | 0.79 | 0.79 | 2.51 | 0.79 | 266.16 | 0.79 | 369.85 | | 0.44 | 0.440 | 0.440 | 0.440 | 0.440 | 0.440 | 0.440 | | 32.6 | 45.3 | 0.16 | 550 | 34.08 | 48.92 |
| 34B | 7/28 | 0.000280 | | 209.66 | 0.79 | 4.71 | 0.79 | 0.79 | 21.20 | 0.79 | 235.57 | | 291.46 | 0.79 | 16.86 | 2.40 | 0.79 | 9.65 | 0.79 | 320.38 | | 0.44 | 0.440 | 20.000 | 0.440 | 0.440 | 0.440 | 20.000 | | 28.0 | 55.4 | 0.06 | 850 | 32.40 | 52.52 |
| 34L | 7/28 | 0.000140 | | 166.97 | 0.79 | 4.71 | 2.40 | 0.79 | 19.08 | 0.79 | 193.17 | | 291.46 | 0.79 | 16.86 | 58.75 | 0.79 | 9.65 | 0.79 | 320.38 | | 0.44 | 0.440 | 20.000 | 0.440 | 0.440 | 0.440 | 20.000 | | 31.1 | 54.3 | 0.03 | 800 | 32.90 | 61.98 |
| 33B | 7/29 | 0.000000 | | 321.84 | 26.64 | 10.66 | 0.79 | 0.79 | 1193.49 | 0.79 | 1571.26 | | 229.13 | 0.79 | 5.24 | 7.29 | 0.79 | 1683.60 | 0.79 | 1938.75 | | 0.44 | 0.440 | 0.440 | 0.440 | 0.440 | 0.440 | 0.440 | | 30.1 | 68.0 | 0.37 | 550 | 30.30 | 70.18 |
| 33L | 7/29 | 0.000000 | | 300.68 | 13.18 | 13.18 | 0.79 | 0.79 | 1075.97 | 0.79 | 1434.63 | | 229.13 | 0.79 | 5.24 | 0.79 | 0.79 | 1683.60 | 0.79 | 1938.75 | | 0.44 | 0.440 | 0.440 | 0.440 | 0.440 | 0.440 | 0.440 | | 26.3 | 85.6 | 0.06 | 450 | 30.01 | 71.45 |
| 49L | 7/29 | 0.000000 | | 427.93 | 19.77 | 9.72 | 7.27 | 0.79 | 51.20 | 0.79 | 515.90 | | 331.09 | 4.89 | 4.71 | 7.29 | 0.79 | 28.94 | 0.79 | 376.91 | | 0.44 | 0.440 | 0.440 | 0.440 | 0.440 | 0.440 | 0.440 | | 31.0 | 69.2 | 0.62 | 450 | 30.77 | 78.91 |
| 08B | 7/30 | 0.000250 | | 372.35 | 2.62 | 608.85 | 10.29 | 0.79 | 0.79 | 0.79 | 994.11 | | 357.81 | 12.85 | 915.25 | 5.02 | 0.79 | 0.79 | 0.79 | 1290.93 | | 16.67 | 0.440 | 0.440 | 0.440 | 0.440 | 0.440 | 16.670 | | 29.1 | 72.3 | 0.00 | 632 | 30.30 | 70.60 |
| 08L | 7/30 | 0.000190 | | 275.35 | 42.64 | 275.52 | 2.49 | 0.79 | 0.79 | 0.79 | 596.00 | | 357.81 | 12.85 | 915.25 | 5.02 | 0.79 | 0.79 | 0.79 | 1290.93 | | 288.33 | 0.440 | 11.670 | 0.440 | 0.440 | 0.440 | 300.000 | | 28.0 | 74.5 | 0.23 | 635 | 28.60 | 73.50 |
| 09B | 7/30 | 0.000000 | | 199.30 | 63.43 | 17.09 | 17.00 | 0.79 | 0.79 | 0.79 | 296.82 | | 270.71 | 21.40 | 0.79 | 0.79 | 0.79 | 0.79 | 0.79 | 292.11 | | 0.44 | 0.440 | 0.440 | 0.440 | 0.440 | 0.440 | 0.440 | | 29.0 | 75.4 | 0.01 | 537 | 31.00 | 67.67 |
| 09L | 7/30 | 0.000000 | | 302.48 | 55.22 | 9.62 | 9.60 | 0.79 | 0.79 | 0.79 | 376.91 | | 270.71 | 21.40 | 0.79 | 0.79 | 0.79 | 0.79 | 0.79 | 292.11 | | 0.44 | 0.440 | 0.440 | 0.440 | 0.440 | 0.440 | 0.440 | | 29.9 | 71.5 | 0.15 | 485 | 30.20 | 72.70 |
| 10B | 7/30 | 0.000000 | | 76.06 | 44.09 | 0.79 | 0.79 | 0.79 | 0.79 | 0.79 | 120.14 | | 206.50 | 90.03 | 9.62 | 4.81 | 0.79 | 0.79 | 0.79 | 310.95 | | 0.44 | 0.440 | 0.440 | 0.440 | 0.440 | 0.440 | 0.440 | | 31.0 | 69.5 | 0.13 | 878 | 30.50 | 64.82 |
| 10L | 7/30 | 0.000000 | | 152.82 | 89.77 | 2.41 | 0.79 | 0.79 | 0.79 | 0.79 | 244.99 | | 206.50 | 90.03 | 9.62 | 4.81 | 0.79 | 0.79 | 0.79 | 310.95 | | 0.44 | 0.440 | 0.440 | 0.440 | 0.440 | 0.440 | 0.440 | | 29.0 | 58.9 | 0.12 | 837 | 31.00 | 55.47 |
| 11B | 8/4 | 0.000000 | | 240.09 | 102.62 | 14.74 | 12.39 | 0.79 | 0.79 | 0.79 | 369.85 | | 341.28 | 125.61 | 63.38 | 65.72 | 0.79 | 0.79 | 0.79 | 596.00 | | 0.44 | 0.440 | 0.440 | 0.440 | 0.440 | 0.440 | 0.440 | | 31.0 | 66.9 | 0.07 | 517 | 32.70 | 65.00 |
| 11L | 8/4 | 0.000000 | | 259.06 | 111.00 | 24.68 | 22.22 | 0.79 | 0.79 | 0.79 | 416.96 | | 341.28 | 125.61 | 63.38 | 65.72 | 0.79 | 0.79 | 0.79 | 596.00 | | 0.44 | 0.440 | 0.440 | 0.440 | 0.440 | 0.440 | 0.440 | | 29.1 | 72.2 | 0.15 | 419 | 32.67 | 65.10 |
| 12B | 8/4 | 0.000370 | | 120.45 | 71.41 | 14.80 | 14.78 | 0.79 | 0.79 | 0.79 | 221.44 | | 121.57 | 72.97 | 2.45 | 29.15 | 0.79 | 0.79 | 0.79 | 226.15 | | 615.00 | 0.440 | 0.440 | 0.440 | 0.440 | 0.440 | 615.000 | | 26.5 | 82.4 | 0.05 | 512 | 30.90 | 64.80 |
| 12L | 8/4 | 0.000000 | | 153.02 | 53.44 | 7.28 | 19.48 | 0.79 | 0.79 | 0.79 | 233.22 | | 121.57 | 72.97 | 2.45 | 29.15 | 0.79 | 0.79 | 0.79 | 226.15 | | 0.44 | 0.440 | 0.440 | 0.440 | 0.440 | 0.440 | 0.440 | | 30.1 | 69.3 | 0.03 | 467 | 31.27 | 61.62 |
| 04B | 8/7 | 0.000000 | | 50.80 | 30.83 | 2.36 | 0.79 | 4.81 | 2.36 | 0.79 | 115.43 | | 36.51 | 24.15 | 2.36 | 0.79 | 23.75 | 0.79 | 0.79 | 96.58 | | 0.44 | 0.440 | 0.440 | 0.440 | 0.440 | 0.440 | 0.440 | | 29.7 | 76.8 | 0.19 | 550 | 30.90 | 69.40 |
| 04L | 8/7 | 0.036430 | | 50.88 | 164.50 | 12.08 | 2.44 | 2.41 | 24.32 | 0.79 | 261.48 | | 114.25 | 96.85 | 0.79 | 0.79 | 2.46 | 0.79 | 0.79 | 228.50 | | 5503.11 | 2734.222 | 0.440 | 6.222 | 0.440 | 82.370 | 8243.556 | | 28.9 | 75.5 | 0.10 | 550 | 31.71 | 84.59 |
| 04L | 8/7 | 0.054310 | | 50.88 | 164.50 | 12.08 | 2.44 | 2.41 | 24.32 | 0.79 | 261.48 | | 114.25 | 96.85 | 0.79 | 0.79 | 2.46 | 0.79 | 0.79 | 228.50 | | 2485.33 | 392.889 | 64.444 | 4072.889 | 0.440 | 247.111 | 7276.000 | | 29.3 | 68.6 | 0.10 | 550 | 31.23 | 79.23 |
| 04L | 8/7 | 0.025450 | | 50.88 | 164.50 | 12.08 | 2.44 | 2.41 | 24.32 | 0.79 | 261.48 | | 114.25 | 96.85 | 0.79 | 0.79 | 2.46 | 0.79 | 0.79 | 228.50 | | 7988.44 | 3127.111 | 64.444 | 4079.111 | 0.440 | 247.111 | 15519.556 | | 30.0 | 72.2 | 0.10 | 550 | 31.59 | 83.25 |
| 25B | 8/10 | 0.000000 | | 82.51 | 12.49 | 0.79 | 0.79 | 0.79 | 413.01 | 0.79 | 522.97 | | 98.32 | 2.40 | 0.79 | 0.79 | 0.79 | 28.67 | 0.79 | 138.99 | | 0.44 | 0.440 | 0.440 | 0.440 | 0.440 | 0.440 | 0.440 | | 27.6 | 61.3 | 0.01 | 900 | 28.34 | 69.90 |
| 25L | 8/10 | 0.000000 | | 93.33 | 4.71 | 0.79 | 0.79 | 0.79 | 141.83 | 0.79 | 266.20 | | 98.32 | 2.40 | 0.79 | 0.79 | 0.79 | 28.67 | 0.79 | 138.99 | | 0.44 | 0.440 | 0.440 | 0.440 | 0.440 | 0.440 | 0.440 | | 28.1 | 79.8 | 0.03 | 600 | 28.90 | 80.03 |
| 27B | 8/10 | 0.000000 | | 88.41 | 40.56 | 14.58 | 0.79 | 0.79 | 0.79 | 0.79 | 148.41 | | 87.71 | 58.26 | 9.42 | 0.79 | 0.79 | 0.79 | 0.79 | 160.19 | | 0.44 | 0.440 | 0.440 | 0.440 | 0.440 | 0.440 | 0.440 | | 29.3 | 79.3 | 0.12 | 550 | 30.03 | 83.12 |
| 27L | 8/10 | 0.000000 | | 48.32 | 58.71 | 27.24 | 0.79 | 0.79 | 0.79 | 0.79 | 136.63 | | 87.71 | 58.26 | 9.42 | 0.79 | 0.79 | 0.79 | 0.79 | 160.19 | | 0.44 | 0.440 | 0.440 | 0.440 | 0.440 | 0.440 | 0.440 | | 28.7 | 83.2 | 0.13 | 500 | 30.30 | 82.74 |
| 02B | 8/11 | 0.000060 | | 2.36 | 0.79 | 0.79 | 0.79 | 0.79 | 0.79 | 0.79 | 2.36 | | 21.20 | 7.07 | 0.79 | 0.79 | 0.79 | 21.20 | 0.79 | 49.47 | | 0.44 | 0.440 | 0.440 | 0.440 | 13.330 | 0.440 | 13.330 | | 27.0 | 56.3 | 0.00 | 1050 | 29.07 | 65.60 |
| 02L | 8/11 | 0.000000 | | 19.63 | 17.16 | 0.79 | 0.79 | 0.79 | 38.59 | 0.79 | 75.38 | | 21.20 | 7.07 | 0.79 | 0.79 | 0.79 | 21.20 | 0.79 | 49.47 | | 0.44 | 0.440 | 0.440 | 0.440 | 0.440 | 0.440 | 0.440 | | 25.1 | 64.3 | 0.33 | 900 | 29.62 | 60.48 |
| 26B | 8/11 | 0.000020 | | 145.42 | 59.15 | 16.86 | 0.79 | 0.79 | 63.62 | 0.79 | 285.04 | | 84.63 | 22.40 | 9.96 | 0.79 | 0.79 | 14.93 | 0.79 | 131.92 | | 0.44 | 0.440 | 0.440 | 0.440 | 686.670 | 0.440 | 686.670 | | 28.6 | 82.3 | 0.11 | 500 | 28.80 | 88.60 |
| 26L | 8/11 | 0.000000 | | 134.06 | 7.33 | 18.02 | 0.79 | 0.79 | 349.43 | 0.79 | 508.83 | | 84.63 | 22.40 | 9.96 | 0.79 | 0.79 | 14.93 | 0.79 | 131.92 | | 0.44 | 0.440 | 0.440 | 0.440 | 0.440 | 0.440 | 0.440 | | 27.0 | 88.8 | 0.12 | 500 | 29.53 | 85.88 |
| 28B | 8/12 | 0.000110 | | 0.79 | 0.79 | 0.79 | 0.79 | 0.79 | 713.78 | 0.79 | 713.78 | | 1.57 | 0.79 | 0.79 | 0.79 | 0.79 | 120.14 | 0.79 | 120.14 | | 0.44 | 0.440 | 0.440 | 0.440 | 3220.000 | 0.440 | 3220.000 | | 29.5 | 75.5 | 0.24 | 450 | 30.10 | 80.57 |
| 28L | 8/12 | 0.000000 | | 0.79 | 0.79 | 2.36 | 0.79 | 0.79 | 275.62 | 0.79 | 277.97 | | 1.57 | 0.79 | 0.79 | 0.79 | 0.79 | 120.14 | 0.79 | 120.14 | | 0.44 | 0.440 | 0.440 | 0.440 | 0.440 | 0.440 | 0.440 | | 29.6 | 74.0 | 0.22 | 450 | 30.40 | 78.67 |
| 30B | 8/12 | 0.000000 | | 50.75 | 0.79 | 0.79 | 14.72 | 0.79 | 33.47 | 0.79 | 98.94 | | 4.71 | 0.79 | 0.79 | 0.79 | 0.79 | 4.71 | 0.79 | 9.42 | | 0.44 | 0.440 | 0.440 | 0.440 | 0.440 | 0.440 | 0.440 | | 28.7 | 85.2 | 0.00 | 600 | 30.02 | 87.58 |
| 30L | 8/12 | 0.000000 | | 207.88 | 0.79 | 0.79 | 22.67 | 0.79 | 278.28 | 0.79 | 508.83 | | 4.71 | 0.79 | 0.79 | 0.79 | 0.79 | 4.71 | 0.79 | 9.42 | | 0.44 | 0.440 | 0.440 | 0.440 | 0.440 | 0.440 | 0.440 | | 28.6 | 84.8 | 0.25 | 550 | 29.43 | 89.93 |
| 44B | 8/24 | 0.000410 | | 744.51 | 51.87 | 14.60 | 14.72 | 0.79 | 17.16 | 0.79 | 855.12 | | 878.03 | 115.03 | 40.81 | 0.79 | 0.79 | 12.82 | 0.79 | 1097.76 | | 0.44 | 0.440 | 0.440 | 0.440 | 10253.330 | 0.440 | 10253.330 | | 28.8 | 79.8 | 0.00 | 800 | 30.60 | 77.63 |
| 44L | 8/24 | 0.000410 | | 777.09 | 106.10 | 25.34 | 14.99 | 0.79 | 30.11 | 0.79 | 961.13 | | 878.03 | 115.03 | 40.81 | 20.46 | 0.79 | 12.82 | 0.79 | 1097.76 | | 0.44 | 10.000 | 0.440 | 0.440 | 9616.667 | 0.440 | 9626.667 | | 28.8 | 79.4 | 0.04 | 750 | 31.10 | 75.20 |
| 45B | 8/24 | 0.000000 | | 120.14 | 0.79 | 7.07 | 0.79 | 0.79 | 18.85 | 0.79 | 157.83 | | 324.87 | 4.90 | 21.77 | 0.79 | 0.79 | 24.31 | 0.79 | 416.96 | | 0.44 | 0.440 | 0.440 | 0.440 | 0.440 | 0.440 | 0.440 | | 29.8 | 70.2 | 0.16 | 1100 | 31.36 | 61.78 |
| 45L | 8/24 | 0.000000 | | 193.19 | 0.79 | 7.24 | 0.79 | 0.79 | 45.86 | 0.79 | 289.75 | | 324.87 | 4.90 | 21.77 | 0.79 | 0.79 | 24.31 | 0.79 | 416.96 | | 0.44 | 0.440 | 0.440 | 0.440 | 0.440 | 0.440 | 0.440 | | 28.4 | 83.7 | 0.23 | 750 | 29.20 | 83.86 |
| 46B | 8/24 | 0.000810 | | 212.53 | 28.96 | 21.76 | 0.79 | 0.79 | 0.79 | 0.79 | 287.40 | | 84.81 | 0.79 | 0.79 | 0.79 | 0.79 | 0.79 | 0.79 | 87.16 | | 36.67 | 0.440 | 0.440 | 0.440 | 8686.667 | 0.440 | 8723.333 | | 29.7 | 71.5 | 0.01 | 1100 | 31.70 | 74.94 |
| 46L | 8/24 | 0.000530 | | 199.75 | 7.13 | 7.13 | 0.79 | 0.79 | 21.38 | 0.79 | 247.35 | | 84.81 | 0.79 | 0.79 | 0.79 | 0.79 | 0.79 | 0.79 | 87.16 | | 0.44 | 0.440 | 0.440 | 0.440 | 1586.670 | 0.440 | 1586.670 | | 30.2 | 70.9 | 0.01 | 1200 | 31.13 | 76.98 |
| 42B | 8/25 | 0.000990 | | 969.47 | 16.79 | 0.79 | 0.79 | 0.79 | 35.96 | 0.79 | 1029.45 | | 52.81 | 0.79 | 2.44 | 0.79 | 0.79 | 305.17 | 0.79 | 360.42 | | 0.44 | 0.440 | 0.440 | 0.440 | 4553.333 | 0.440 | 4553.333 | | 30.2 | 73.5 | 0.29 | 850 | 30.90 | 77.13 |
| 42L | 8/25 | 0.000290 | | 242.61 | 19.76 | 0.79 | 0.79 | 0.79 | 347.65 | 0.79 | 612.49 | | 52.81 | 0.79 | 2.44 | 0.79 | 0.79 | 305.17 | 0.79 | 360.42 | | 0.44 | 0.440 | 0.440 | 0.440 | 3860.000 | 0.440 | 3860.000 | | 30.3 | 75.6 | 0.35 | 1200 | 31.00 | 80.76 |
| 48B | 8/25 | 0.001410 | | 242.00 | 11.84 | 2.36 | 0.79 | 0.79 | 19.16 | 0.79 | 294.46 | | 277.60 | 7.07 | 9.46 | 0.79 | 0.79 | 11.94 | 0.79 | 320.38 | | 0.44 | 0.440 | 0.440 | 0.440 | 3480.000 | 0.440 | 3480.000 | | 28.3 | 76.4 | 0.09 | 950 | 30.04 | 75.26 |
| 48L | 8/25 | 0.001160 | | 283.95 | 7.22 | 4.83 | 0.79 | 0.79 | 9.64 | 0.79 | 334.51 | | 277.60 | 7.07 | 9.46 | 20.46 | 0.79 | 11.94 | 0.79 | 320.38 | | 0.44 | 0.440 | 0.440 | 0.440 | 3813.330 | 0.440 | 3813.330 | | 28.5 | 78.2 | 0.05 | 1000 | 29.68 | 81.04 |
| 20B | 8/31 | 0.000250 | | 223.42 | 0.79 | 0.79 | 7.19 | 7.13 | 11.96 | 0.79 | 249.71 | | 89.52 | 2.36 | 0.79 | 12.22 | 21.20 | 0.79 | 0.79 | 124.85 | | 0.44 | 0.440 | 0.440 | 0.440 | 796.670 | 0.440 | 796.670 | | 31.7 | 65.5 | 0.14 | 1100 | 31.40 | 72.70 |
| 20L | 8/31 | 0.000000 | | 137.98 | 7.24 | 0.79 | 0.79 | 50.31 | 2.36 | 0.79 | 197.88 | | 89.52 | 2.36 | 0.79 | 11.78 | 21.20 | 0.79 | 0.79 | 124.85 | | 0.44 | 0.440 | 0.440 | 0.440 | 0.440 | 0.440 | 0.440 | | 30.5 | 70.0 | 0.27 | 900 | 31.58 | 73.08 |
| 23B | 8/31 | 0.000140 | | 124.36 | 136.34 | 4.83 | 4.94 | 0.79 | 14.58 | 0.79 | 285.04 | | 117.57 | 44.07 | 7.35 | 0.79 | 0.79 | 4.90 | 0.79 | 186.10 | | 0.44 | 0.440 | 0.440 | 0.440 | 3993.330 | 0.440 | 3993.330 | | 29.8 | 71.3 | 0.19 | 850 | 30.16 | 76.96 |
| 23L | 8/31 | 0.000000 | | 125.11 | 91.36 | 2.41 | 0.79 | 0.79 | 9.62 | 0.79 | 228.50 | | 117.57 | 44.07 | 7.35 | 12.22 | 0.79 | 4.90 | 0.79 | 186.10 | | 0.44 | 0.440 | 0.440 | 0.440 | 0.440 | 0.440 | 0.440 | | 30.1 | 68.8 | 0.45 | 800 | 31.04 | 71.56 |
| 24B | 8/31 | 0.000090 | | 9.42 | 0.79 | 0.79 | 0.79 | 4.71 | 63.60 | 0.79 | 77.74 | | 229.16 | 33.90 | 2.46 | 11.78 | 9.67 | 115.68 | 0.79 | 407.54 | | 0.44 | 0.440 | 0.440 | 0.440 | 1340.000 | 0.440 | 1340.000 | | 30.9 | 65.0 | 0.00 | 1000 | 31.23 | 37.92 |
| 24L | 8/31 | 0.000020 | | 119.41 | 19.01 | 7.15 | 19.09 | 11.78 | 21.45 | 0.79 | 197.88 | | 229.16 | 33.90 | 2.46 | 16.68 | 9.67 | 115.68 | 0.79 | 407.54 | | 0.44 | 0.440 | 0.440 | 0.440 | 340.000 | 0.440 | 340.000 | | 31.6 | 60.8 | 0.05 | 750 | 31.20 | 46.60 |
| 17B | 9/1 | 0.000000 | | 119.15 | 85.41 | 4.86 | 7.22 | 7.14 | 0.79 | 0.79 | 226.15 | | 84.44 | 108.52 | 7.22 | 16.68 | 0.79 | 0.79 | 0.79 | 202.59 | | 0.44 | 0.440 | 0.440 | 0.440 | 0.440 | 0.440 | 0.440 | | 29.7 | 75.6 | 0.01 | 550 | 31.26 | 65.90 |
| 17L | 9/1 | 0.000000 | | 104.84 | 80.20 | 0.79 | 9.71 | 9.78 | 0.79 | 0.79 | 216.73 | | 84.44 | 108.52 | 7.22 | 0.79 | 0.79 | 0.79 | 0.79 | 202.59 | | 0.44 | 0.440 | 0.440 | 0.440 | 0.440 | 0.440 | 0.440 | | 30.4 | 77.4 | 0.04 | 450 | 31.24 | 66.08 |
| 19B | 9/1 | 0.000000 | | 124.84 | 36.61 | 7.32 | 19.47 | 0.79 | 7.29 | 0.79 | 195.52 | | 124.98 | 48.68 | 7.35 | 35.33 | 0.79 | 0.79 | 0.79 | 202.59 | | 0.44 | 0.440 | 0.440 | 0.440 | 0.440 | 0.440 | 0.440 | | 26.3 | 72.6 | 0.16 | 700 | 31.42 | 55.72 |
| 19L | 9/1 | 0.000000 | | 161.60 | 35.90 | 2.45 | 11.87 | 0.79 | 9.61 | 0.79 | 221.44 | | 124.98 | 48.68 | 7.35 | 21.58 | 0.79 | 0.79 | 0.79 | 202.59 | | 0.44 | 0.440 | 0.440 | 0.440 | 0.440 | 0.440 | 0.440 | | 29.7 | 73.8 | 0.13 | 450 | 33.38 | 62.98 |
| 21B | 9/1 | 0.000920 | | 75.16 | 55.57 | 7.23 | 14.46 | 21.72 | 0.79 | 0.79 | 181.39 | | 109.08 | 206.47 | 15.22 | 0.79 | 101.91 | 5.09 | 0.79 | 506.48 | | 0.44 | 0.440 | 0.440 | 0.440 | 3066.670 | 0.440 | 3066.670 | | 29.5 | 68.0 | 0.22 | 500 | 32.07 | 58.93 |
| 21L | 9/1 | 0.000350 | | 95.17 | 127.61 | 7.48 | 44.82 | 73.13 | 0.79 | 0.79 | 355.71 | | 109.08 | 206.47 | 15.22 | 35.33 | 101.91 | 5.09 | 0.79 | 506.48 | | 0.44 | 0.440 | 0.440 | 0.440 | 60.000 | 0.440 | 60.000 | | 31.3 | 68.4 | 0.30 | 450 | 31.64 | 67.08 |
| 18B | 9/2 | 0.000000 | | 68.32 | 9.42 | 11.78 | 14.13 | 0.79 | 4.71 | 0.79 | 108.36 | | 97.85 | 56.28 | 14.68 | 21.58 | 0.79 | 0.79 | 0.79 | 190.81 | | 0.44 | 0.440 | 0.440 | 0.440 | 0.440 | 0.440 | 0.440 | | 29.1 | 72.2 | 0.03 | 550 | 29.06 | 74.16 |
| 18L | 9/2 | 0.000000 | | 110.14 | 14.69 | 22.03 | 39.14 | 0.79 | 2.45 | 0.79 | 188.46 | | 97.85 | 56.28 | 14.68 | 22.00 | 0.79 | 0.79 | 0.79 | 190.81 | | 0.44 | 0.440 | 0.440 | 0.440 | 0.440 | 0.440 | 0.440 | | 29.2 | 68.3 | 0.23 | 500 | 29.96 | 69.34 |
